# Real‐World Databases in Diabetes: A Systematic Review

**DOI:** 10.1002/dmrr.70241

**Published:** 2026-10-16

**Authors:** Jonah J. C. Thomas, Anna Meffen, Alessandro Rizzi, Andrew P. Kingsnorth, Beatrice Pieri, Ying He, Shirley Konnor, Clare Gillies, Kamlesh Khunti, Jonathan Valabhji, Shivani Misra, Amanda Adler, Christian Torp‐Pedersen, Martina Vettoretti, Julia K. Mader, Pratik Choudhary

**Affiliations:** ^1^ University of Leicester University Road Leicester UK; ^2^ Leicester Diabetes Centre Gwendolen Road Leicester UK; ^3^ Diabetes Care Unit Università Cattolica del Sacro Cuore Fondazione Policlinico Agostino Gemelli IRCCS Rome Italy; ^4^ School of Sport, Exercise and Health Sciences Loughborough University Loughborough UK; ^5^ NIHR Leicester Biomedical Research Centre Leicester UK; ^6^ Department of Mathematics and Statistics University of Strathclyde Glasgow UK; ^7^ Department of Metabolism, Digestion & Reproduction Faculty of Medicine, Imperial London UK; ^8^ Department of Diabetes and Endocrinology Chelsea and Westminster Hospital NHS Foundation Trust London UK; ^9^ Department of Diabetes & Endocrinology Imperial College Healthcare NHS Trust London UK; ^10^ Diabetes Trials Unit Radcliffe Department of Medicine University of Oxford Oxford UK; ^11^ Copenhagen University Hospital Steno Diabetes Center Copenhagen Herlev Denmark; ^12^ Department of Information Engineering University of Padova Padova Italy; ^13^ Division of Endocrinology and Diabetology Department of Internal Medicine Medical University of Graz Graz Austria

**Keywords:** database characteristics, meta data, real‐world evidence

## Abstract

**Aim:**

To identify publications that describe RWD databases in the field of diabetes and map their key characteristics.

**Methods:**

Databases were searched (MEDLINE, SCOPUS, CINAHL from January 2018 to October 2025) to identify relevant publications describing RWD databases. Eligible studies were identified as RWD databases that contained more than 1000 adults and were followed up for one year or longer. Metadata including population, sample size, and outcomes were then extracted.

**Results:**

A total of 610 eligible RWD databases were identified from 1779 included articles. Databases were identified in 74 countries, with the majority collected in Europe (*n* = 193). Only 22 databases were identified in lower‐ and‐ middle income countries (as defined by the World Bank). Demographics, prescription, diagnosis, and biochemical information were collected frequently; however, mortality and technology related variables (CGM usage) were found in fewer datasets. Very few databases were open access however 205 mentioned having the possibility for data sharing.

**Conclusion:**

We have produced an online dashboard to summarise the RWD databases available in the field of diabetes. We found many RWD databases globally that contain a range of variables related to individuals' health; however, lower‐ and middle‐income nations are underrepresented within these data.

## 1 Introduction

Randomised controlled trials (RCTs) are considered the gold standard for examining the impact of a given treatment on an outcome [1] as the process of randomisation reduces bias allowing the causal relationship between a treatment and outcome to be assessed [2]. Currently, RCTs, more particularly meta‐analysis of multiple RCTs, are the favoured highest level of evidence to inform decision making regarding the safety, efficacy, and cost‐effectiveness of treatments.

Real‐world data (RWD) can provide complementary evidence to assess the effectiveness, and safety, of a treatment and may have utility in addressing some of the key limitations of RCT‐derived evidence [3]. These limitations include 1) the considerable time and financial burden of undertaking an RCT; 2) the lack of diversity in age, gender, ethnicity and deprivation of RCT participants, which limits generalisability to the wider population; 3) scenarios in which conducting an RCT may be deemed unethical due to known patient benefit and 4) the highly regulated way in which they are conducted, participants are monitored and overseen is somewhat artificial compared to real‐world usage. Taken together, some of these limitations are likely to account for the efficacy‐to‐effectiveness gap between trial evidence and real‐word effectiveness [4].

RWD has previously been defined by the US Food and Drug Administration as ‘data relating to patient health status and/or the delivery of health care routinely collected from a variety of sources’ [5]. Several key characteristics of RWD have been identified, including being observational in nature, being unstructured, being collected at high frequency (minute by minute wearable data) producing large datasets, and being incomplete or inconsistent [3].

Increasingly, RWD is being sought to complement the findings of RCTs in regulatory decision making [6]. RWD can provide evidence of the effectiveness, and safety, of therapies in the real‐world rather than in the highly controlled conditions of an RCT. Further, RWD can be used to answer a diverse range of research questions in areas such as comparative effectiveness research, patterns of drug utilisation, and population surveillance [7].

Patients in real‐world datasets are not randomized nor are all treatment decisions protocolised; however, the large number of patients in these datasets has the potential to overcome some of the uncertainty introduced from variable clinical decision‐making and offers the potential to derive new evidence. Moreover, RWD has the potential to ensure greater diversity in patient characteristics; currently, most RCTs are conducted in high‐income countries and representative patient data from low‐and‐middle income countries (LMICs) and across ethnic groups in diabetes is severely lacking [8]. The paucity of RCT data from certain populations does not provide evidence of the effectiveness of therapies within these populations.

In the field of diabetes, RWD is available from a number of sources such as electronic health records, insurance claims data, and wearable devices [3]. Several initiatives are currently exploring the potential role of RWD in different disease areas such as diabetes (REDDIE) [9], cardiovascular disease (CVD‐REAL) [10], and cancer (ONCOVALUE) [11]. In this systematic review, we aimed to identify RWD databases in the field of diabetes, map their metadata including size and regional coverage and provide a comprehensive assessment of available RWD globally for researchers.

## 2 Materials and Methods

The protocol for this systematic review was uploaded to Open Science Framework (OSF) (https://osf.io/87t5z/ and Electronic Supporting Information S1: Material 1) and is reported in line with the preferred reporting items for systematic reviews and meta‐analyses (PRISMA) statement (Electronic Supporting Information S1: Material 2) [12].

### 2.1 Eligibility Criteria

Eligibility criteria were applied to both the manuscripts and the datasets analysed within them. This review focuses on databases with a sufficient number of adults, with sufficient duration of follow‐up, to make meaningful conclusions from the databases. We therefore decided to focus on databases with at least 1000 individuals and at least 12 months of follow‐up. Studies were required to be published in the last 5 years to limit the number of now discontinued databases. To ascertain the breadth of available RWD globally, we included all datasets from the same country even if it was likely that more than one of them shared individual patient data. Exclusion criteria included datasets focused on gestational diabetes, being cross‐sectional in nature (did not include follow‐up data), and being a systematic review or meta‐analyses. A full list of inclusion and exclusion criteria is provided in Table 1.

**TABLE 1:** Eligibility criteria for each dataset.

Inclusion	Exclusion
Describe or analyse a RWD database which focuses on adult patients with diabetes^a^	Focus on paediatric or gestational population
Observational, longitudinal study design; or include routinely collected health data	Is a systematic review or protocol paper with no data^a^
Contain data on at least 1000 participants living with diabetes	
Include a follow‐up duration of at least 12 months	
Published in last 5 years^a^	

^a^ Criteria applied to the publication, not the dataset.

### 2.2 Searches

Searches were conducted in three electronic databases (Ovid Medline, SCOPUS, CINAHL) from January 1st, 2018, to October 2nd, 2023. An updated search was performed in Ovid Medline from October, 2023, to, October 2025.

Search terms focused on diabetes, outcomes, and study design. Reference lists of the included studies were also searched. The search strategy is included in Electronic Supporting Information S1: Material 3.

### 2.3 Data Screening

Due to the large number of results returned, a semi‐automated screening process was conducted to reduce the number of articles which required title and abstract screening using custom written R code. To do this, numbers within the abstract were extracted and only abstracts with a sample size greater than 1000 were included. Additionally, abstracts with the terms ‘thousands’ or ‘millions’ were also included to keep studies which did not express their sample size numerically. The code used to complete this screening can be found on GitHub (https://github.com/jonahthomas/REDDIE_RWD_Review/blob/main/autoamated_screening.R).

After duplicates were removed, title and abstract screening was performed. All studies were screened by one researcher (JJCT), with second screening performed by one of several researchers (Alessandro Rizzi, Andrew P. Kingsnorth, Anna Meffen, Ying He, Shirley Konnor, Beatrice Pieri). Any conflicts were resolved by discussion between researchers. After this, full‐text screening was performed by JJCT.

### 2.4 Variable Extraction and Synthesis

Data extraction was performed in two stages. Firstly, data were extracted from the paper, including the year of publication, corresponding author, and dataset analysed. Then, dataset extraction was performed on all identified datasets to identify metadata related to them using a standardised form. All data extraction was performed by a single researcher (JJCT). Several variables were extracted regarding each dataset including the country of data collection, years of data collection, number of participants, outcomes collected, demographic data (including sex, age, ethnicity), prescription data (any medications that were prescribed), diagnosis (include ICD‐10 codes or other clinical diagnosis information), mortality, biochemical data (HbA1c, blood lipids), technology (use of continuous glucose monitoring (CGM), insulin pumps), and open access status of the database. To extract these data, we used cohort profile publications, web searches for database websites, and all identified publications related to that database as required. The criteria used to determine each of the above are described in detail in Electronic Supporting Information S1: Material 4.

A narrative summary of the data extracted is presented in this paper. Further, an online dashboard was created to allow users to identify RWD databases in diabetes, which may be best suited to answer their specific research questions (https://diabetes‐review‐dashboard.netlify.app).

## 3 Results

A total of 15,380 articles (2329 from the updated search) were title and abstract screened after automated screening and duplicates were removed. From this, 1759 were full‐text screened with 1216 relevant articles identified. From these articles, 610 unique global datasets were extracted (Electronic Supporting Information S1: Material 5). A PRISMA diagram outlining the screening process is provided in Figure 1 below.

**FIGURE 1:**
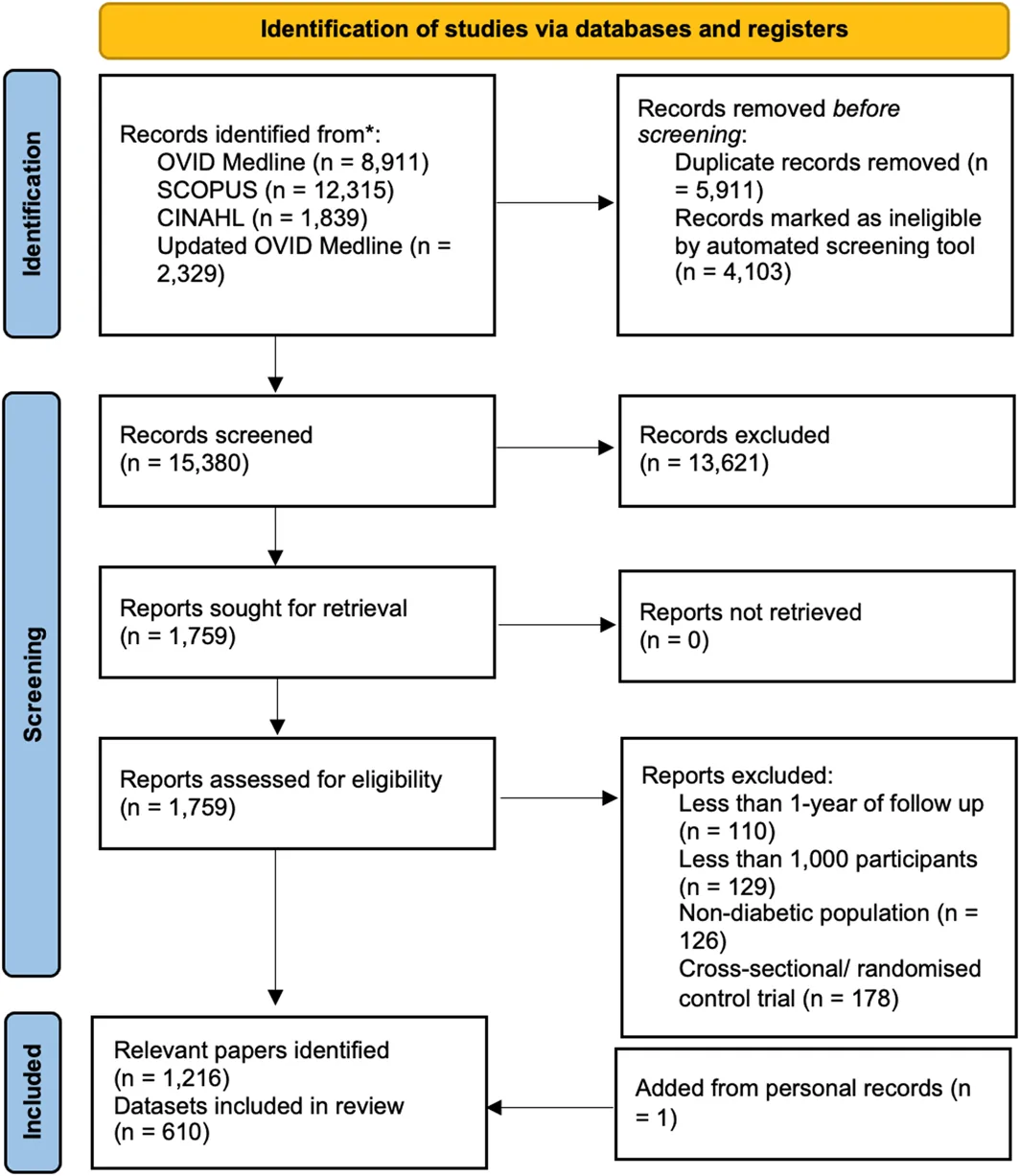
PRISMA diagram showing the current progress of the systematic review.

Of the databases extracted, 315 (52%) included individuals living with type 2 diabetes (T2D), 22 (3.6%) with type 1 diabetes (T1D), and 265 (44%) with any type of diabetes. Registry data constituted 242 datasets (40%), 207 (34%) were from electronic medical record (EMRs), and 64 (11%) were from claims databases. We also identified 95 (16%) databases collected from research studies. The median number of patients in each database was 12,418 (range: 1000 to 246,558,805). A summary of the metadata collected from the datasets identified is included in Table 2 below.

**TABLE 2:** Summary of the metadata collected from the datasets identified.

Variable	Number of datasets (%) [total number of individuals^a^]
Datasets	610 [556,886,910^a^]
Region of data collection	
Africa‐total	4 (0.7) [63,636]
South Africa	2 (0.3)
Democratic republic of the Congo	1 (0.2)
Asia‐total	186 (30) [42,684,693]
Japan	53 (8.7)
China	35 (5.7)
Europe‐total	193 (32) [41,062,917]
United Kingdom	30 (4.9)
Italy	25 (4.1)
North America‐total	180 (30) [215,847,224]
United States	148 (24)
Canada	32 (5.3)
South America‐total	5 (0.8) [1,139,924]
Brazil	3 (0.5)
Colombia & Mexico	1 (0.2)
Oceania‐total	22 (3.6) [2,134,652]
Australia	16 (2.6)
New Zealand	6 (1.0)
Population	
Diabetes	266 (44) [262,133,367]
T1D	22 (3.6) [288,726]
T2D	315 (52) [294,211,780]
Database type	
Registry	242 (40) [91,010,452]
Claims database	64 (11) [17,713,223]
EMRs	208 (34) [442,333,464]
Study	95 (16) [5,829,771]
Accessibility	
Openly available	8 (1.3) [9,171,048]
On request	205 (33) [243,540,505]
Not available	43 (7.1) [6,714,804]
Unknown	352 (58) [296,127,596]
Variables collected	
Patient demographics	591 (97) [551,830,289]
Prescription	508 (83) [546,253,481]
Diagnosis	454 (74) [524,649,940]
Mortality	190 (31) [176,643,886]
Biochemistry	414 (68) [359,227,837]
Technology	37 (6.1) [22,097,078]

*Note:* On request: the dataset can be requested from study authors or applied for through a data access process. Diabetes refers to databases which contain individuals living with both type 1 and type 2 diabetes.

Abbreviations: T1D, type 1 diabetes; T2D, type 2 diabetes.

^a^ The total number of individuals suffers from double counting (the same participant included in two datasets) and therefore does not represent the unique number of individuals.

### 3.1 Dataset Size and Numbers

The majority of datasets were collected within a single country; however, 25 (4%) real‐world databases were collected across multiple countries. Europe had 192 (32%) real‐world datasets, North America 180 (30%), and Asia 185 (30%), with fewer databases collected in Oceania (*n* = 22 (4%)), Africa (*n* = 4 (0.7%)) and South America (*n* = 5 (0.8%)). The United States had the most datasets (*n* = 148 (24%)), followed by Japan (*n* = 53 (9%)), Canada (*n* = 32 (5%)) and the United Kingdom (*n* = 30 (5%)). Of the datasets identified, 22 were collected in LMIC nations including Indonesia, Iran, and Brazil. Figure 2 below shows the number of datasets present globally in the datasets.

**FIGURE 2:**
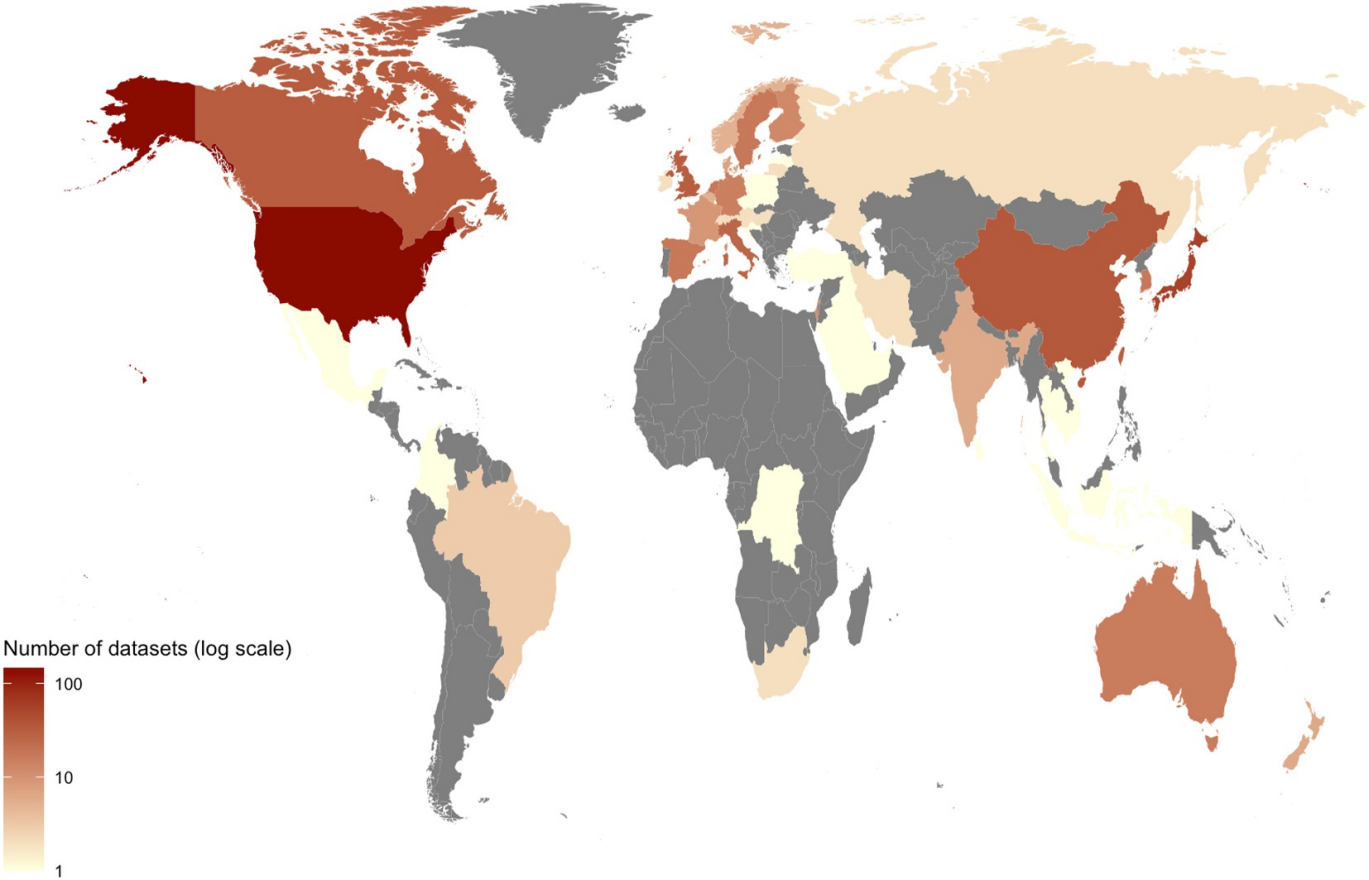
The spread of datasets across countries. Lighter colours represent a smaller number of datasets on a logarithmic scale.

### 3.2 Data Collected

Within the databases identified, patient demographic information was collected in most databases (591/610, 97%). Medication prescription data were collected in 508 datasets (83%), diagnosis data in 454 datasets (74%), and a range of biochemical data in 414 datasets (68%). Mortality data were less commonly collected, being reported in only 190 datasets (31%) and few databases collected data on diabetes technology usage (37, 6%).

Figure 3 shows the number of datasets which collected each of the groups of variables stratified by region.

**FIGURE 3:**
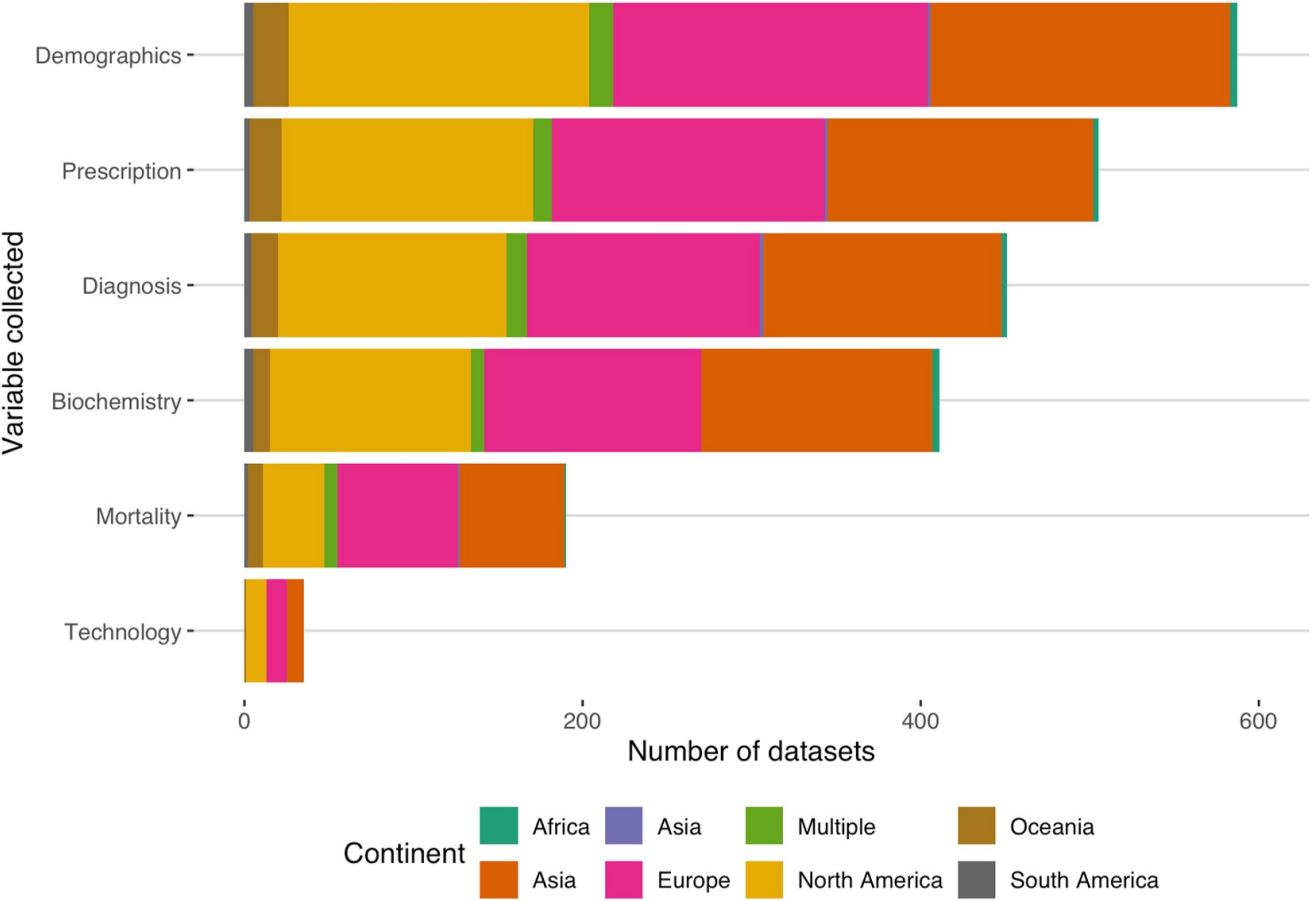
The number of datasets which have collected each group of variables by region.

## 4 Discussion

This systematic review aimed to identify publications describing RWD databases of people living with diabetes globally. We identified 610 databases across five continents, which varied significantly in the number of individuals they included as well as the variables collected. These findings highlight the breadth of RWD that are available to researchers to help address evidence‐gaps. However, there is an evident paucity of data in LMIC nations, suggesting a lack of geographic representation in RWD. Many datasets have measured demographics, prescription, diagnosis, and biochemical data; however, few have collected data on mortality or technology variables.

### 4.1 Previous Literature

A previous systematic review identified national diabetes registries. Bak et al. identified 12 databases comprising 7,181,356 patients [13]. The study reported that nearly all registries included data on weight, HbA1c, lipid profile, and insulin treatment; however, the collection of other variables varied greatly. This finding appears consistent with the databases we identified; biochemical (HbA1c, lipids, etc.) and insulin treatment (prescription data) were commonly reported, whereas other variables, such as mortality, were less commonly collected. Our review extends this work by not only including national registries but also all datasets with over 1000 participants and over one year of follow‐up.

### 4.2 Variable Collection

Demographics, prescription, diagnosis, and biochemical data were collected in a large majority of the databases identified as part of this review. However, far fewer collected data on mortality. Only 34 (6%) databases included technology variables. This is likely due to diabetes technology still being emerging in some nations whilst it is well established in higher income countries. However, whilst technology is becoming established in care guidelines, the usage of such technologies is currently not routinely collected in RWD even in higher income countries. Also, linking of data from wearables such as activity monitors or continuous glucose monitors to electronic health records is not routine; however, this is likely to change in the coming years. Therefore, it can be expected that the number of databases which contain CGM, insulin pumps, and other wearable data is likely to increase over the next decade.

### 4.3 Geographic Distribution

This review highlights that there is an enormous amount of RWD available in individuals living with diabetes; however, it also shows large areas of the world where there is a dearth of data. Most diabetes datasets were available in Europe, North America, and Asia, with significantly fewer collected in Oceania, South America, and Africa. The largest proportion of datasets were collected in the United States, Japan, and the United Kingdom. Datasets were identified in 74 countries showing the wide coverage of RWD databases in the field of diabetes. Within these countries, a variety of health care systems are in place, meaning comparison is possible across databases. The majority of datasets were collected in higher income countries with only 22 collected in LMICs, likely reflecting resources available to collect EMRs. It should be noted that many LMICs have excellent infrastructure in place for communicable disease surveillance, including digital records, which could be leveraged in such countries for non‐communicable diseases (NCDs).

The paucity of data in regions such as Sub‐Saharan Africa highlights that like RCT evidence, RWD may also lack representativeness across all regions and ethnic groups. This presents challenges to researchers who may want to answer questions regarding specific population areas not currently captured. Considering that over 80% of the world population living with T2D reside in LMIC nations [14], the lack of evidence available in these nations means greater efforts to capture these data from LMICs should be explored. Mobile phones may offer a potential solution to capture data at scale [15]. Arguably, it may be easier and have greater long‐term benefit to develop RWD rather than conducting and replicating RCTs in these areas.

### 4.4 Access Status

Only eight identified databases were openly available for analysis. As these databases contain potentially identifiable health data on patients it is to be expected that fully open sharing of data will not be possible. Of the remaining databases, 205 were available on request (it was not clear whether these request processes were associated with a cost) or following a form of application process. It was worth noting the resolution of data available was not possible to ascertain, but given GDPR restrictions, it is likely that only summary data could be shared. However, 43 databases stated it was not possible for them to be shared with other researchers and for 352 the access status could not be ascertained. With many journals now expecting data access statements to be included in manuscripts, it is surprising that the access status could be retrieved for over half of the databases identified. Further, a review found that only 14% of the papers they identified had data access statements, and only 6.8% of the authors responded with the requested data [16]. Therefore, even though many databases state that they are available on request, this may not mean accessing the data is a simple or quick process.

### 4.5 Next Steps

An online dashboard has been produced to summarise and showcase the results of this review. It can be found at https://diabetes‐review‐dashboard.netlify.app. This dashboard allows researchers to examine the key results of this review as well as a database of each dataset identified as part of this review.

### 4.6 Strengths and Limitations

A key strength of this review is the breadth of RWD databases which have been identified in publications. This was achieved by using a wide search strategy, utilised across multiple global databases, which identified a large number of relevant studies. Whilst this limited the number of metadata that could be extracted regarding each database, it allowed our review to capture as comprehensively as possible the available RWD databases across the globe.

A limitation of this review is that only information regarding a database which has been published can be reported. Therefore, some databases may contain more patients or have collected additional variables that are not reported in this review. It is also likely that the number of patients reported in each database is not exclusive, meaning that some patients may appear in multiple databases. For example, if a country has both a diabetes registry as well as a prescription claim database, it is possible that the same individual may be double counted and appear in both. This concept has been previously recognised and is explored in detail by Hussein and colleagues [17]. Due to the large number of studies identified, a semi‐automated abstract screening was performed. This could have led to a small number of datasets being excluded, which may have fulfilled the inclusion criteria. Furthermore, the large number of identified databases meant that data was only extracted by one researcher rather than double extracted. We recognise that a key limitation of this review is that it requires continual updating to ensure it has the highest possible utility to other researchers and key stakeholders. There is currently no one method which has been decided to accomplish this; however, we plan to engage in further discussions to explore available options. If you believe a dataset is suitable for inclusion in this review but is not currently available, please email the corresponding author. The dataset will be reviewed and if it meets the inclusion criteria, it will be added to the tool.

## 5 Conclusion

This systematic review identified 610 RWD databases in the field of diabetes and mapped their characteristics. Datasets were collected across 74 countries across all five continents; however, RWD from certain regions such as Africa and LMICs were underrepresented in terms of the number of datasets identified. Demographics, prescription, diagnosis, and biochemical variables were commonly collected, whereas mortality and technology variables were less collected. This resource can help researchers identify appropriate RWDs to answer future research questions. While RWD has huge potential to address the limitations of RCT‐derived evidence, establishing the infrastructure to collect RWD in all countries should be deemed a priority.

## Author Contributions

J.J.C.T. produced the study methodology, completed data curation, formal analysis, and wrote the original draft. A.M., A.R., A.P.K., B.P., Y.H., S.K. assisted with data curation. C.G., K.K., J.V., S.M. helped produce the study methodology and conceptualised the study. A.A., C.T.P., M.V., J.K.M. all contributed to conceptualising the study. P.C. conceptualised the study and assisted with methodology. All authors reviewed and edited the final manuscript.

## Funding

Funded by the European Union under contract number 101095556. Views and opinions expressed are however those of the author(s) only and do not necessarily reflect those of the European Union or European Health and Digital Executive Agency (HADEA). Neither the European Union nor the granting authority can be held responsible for them. This work has received funding from the UK Research and Innovation.

## Conflicts of Interest

K.K. has acted as a consultant or speaker or received grants for investigator‐initiated studies for Astra Zeneca, Bayer, Novo Nordisk, Sanofi‐Aventis, Servier, Lilly and Merck Sharp & Dohme, Boehringer Ingelheim, Oramed Pharmaceuticals, Pfizer, Roche, Daiichi‐Sankyo, Applied Therapeutics, Embecta and Nestle Health Science. K.K. is Chair of the NDA Research Steering Group. J.K.M. is a member of advisory boards of Abbott Diabetes Care, Becton‐Dickinson, Biomea Fusion, Dexcom, Eli Lilly, Embecta, Medtronic, myLife, Novo Nordisk A/S, Pharmasens, Roche Diabetes Care, Sanofi‐Aventis, Tandem, Viatris and received speaker honoraria from A. Menarini Diagnostics, Abbott Diabetes Care, Dexcom, Eli Lilly, Medtrust, MSD, Novo Nordisk A/S, Roche Diabetes Care, Sanofi, Viatris and Ypsomed. She is a shareholder of decide Clinical Software GmbH and elyte Diagnostics and serves as CMO of elyte Diagnostics. A.A. has received travel support from Lilly related to a trial executive committee. J.V. was the National Clinical Director for Diabetes and Obesity at NHS England from April 2013 to September 2023. P.C. has acted as a consultant for Cambridge Mechatronics Ltd, vTv Therapeutics. Tandem and MyLife Diabetes Care. Consultant, advisory board member and speaker for Medtronic, Novo Nordisk, Sanofi, Lilly, Abbott, Insulet, AstraZeneca, Dexcom, Glooko and Roche. Advisory board member for Ypsomed, Embecta, Tandem and Vertex. He has received research support from Medtronic, Abbott, Novo Nordisk, Dexcom, Roche and Insulet.

## Supporting information


Supporting Information S1

Supporting Information S2

## Data Availability

All data related to this publication can be found within the manuscript or Electronic Supporting Information S1. The code used to perform semi‐automated screening can be found at https://github.com/jonahthomas/REDDIE_RWD_Review/blob/main/autoamated_screening.R.
