# Supplementary material for: Real‐World Databases in Diabetes: A Systematic Review
Source: Diabetes Metab Res Rev. 2026 Oct 16;42(7):e70241. doi: 10.1002/dmrr.70241 (PMC13673215; doi:10.1002/dmrr.70241)
Supplement: Supplementary file 1 [file DMRR-42-e70241-s001.docx]

Electronic Supplementary Material 1: Systematic Review Protocol:

**Real-world Databases in the field of diabetes: a systematic review**

**A systematic review protocol.**

This systematic review protocol registration is based on a modified version of the PROSPERO systematic review registration form. This protocol is registered with the Open Science Framework.

**Introduction**

Diabetes mellitus is a major and growing public health concern. By 2030, it is expected that over 10% of the adult global population will have diabetes costing $125 billion (1). In Europe, 60 million individuals are currently diagnosed with diabetes with a further 36 million being categorised as at risk. Randomised controlled trials (RCTs) have long been regarded as crucial evidence in assessing the effectiveness of clinical interventions across many conditions including diabetes (2). However, with the high costs and recognised limitations of RCTs increasing attention has been placed on real-world data (RWD).

RWD has been defined by the US Food and Drug Administration as “data relating to patient health status and/or the delivery of health care routinely collected from a variety of sources” (3). With the increasing use of electronic means of data collection, combined with the increased ability to store large quantities of data, sources of RWD have increased. Electronic health records, insurance/billing claims, disease registries, as well as mobile and wearable technologies are all potential sources of RWD. RWD can be used to confirm, complement, and augment the findings of RCTs whilst in some cases being able to explore the efficacy, rather than the effectiveness, of a given medical therapy. Further, RWD may be able to help answer research questions which may be unethical, on unfeasible, to answer with a RCT. RWD is particularly relevant to long-term conditions, such as diabetes, where many factors such as drugs, lifestyle interventions and technologies may interact.

As the use of RWD increases, ensuring that the RWD databases available are suitable to answer the specific research question is a key step in effectively generating real-world evidence (RWE). In 2021, the Assessment of Real-World Observational Studies (ArRoWS) criteria was developed to assess the quality of studies which have utilised real-world studies (4). Whilst this tool is useful to help assess studies after they have been published, to date no similar criteria have been published to allow researchers to assess which RWD databases may be most suitable to answer their specific research question. With the growing number of RWD databases available to researcher, and the variation present within these databases, makes choosing which database(s) to utilise a key question.

Therefore, this systematic review aims to identify RWD databases within diabetes and from these, derive a list of considerations when assessing the “fit-for-purpose” of RWD databases for specific research questions.

1. Anticipated or actual start date

01/08/2023

2. Anticipated end date

31^st^ December 2023

3. Stage of review at time of submission

| **Review Stage** | **Started** | **Completed** |
| --- | --- | --- |
| Preliminary searches | Y | N |
| Piloting of study selection process | Y | N |
| Formal screening of search results against eligibility criteria | N | N |
| Data extraction | N | N |

4. Named contact

Mr Jonah Thomas

5. Contact email

jjct1@leicester.ac.uk

6. Contact address

Gwendolen Rd, Leicester LE5 4PW

7. Organisational affiliation of review

Leicester Diabetes Centre

8. Review team members and their organisational affiliations

Mr Jonah Thomas, Leicester Diabetes Centre

Professor Pratik Choudhary, Leicester Diabetes Centre

9. Funding sources/sponsors

HORIZON-HLTH-2022-TOOL-11-02 grant number 101095556

10. Conflict of interest

None to declare.

11. Collaborators

None on this specific work package, please see a full list of REDDIE collaborators here: <https://www.reddie-diabetes.eu/>

12. Review question

This review aims to identify real-world databases in diabetes. To achieve this, three specific research questions were derived:

a) To identify real-world datasets in diabetes which have measured a range of demographic, drug therapy, lifestyle, and outcome variables such as mortality or hospital admissions.
b) To extract meta-data (i.e. information on the variables included) from each identified database including demographics, biochemistry, multi-morbidities, therapies, lifestyle questions, glucose data including continuous glucose monitoring, and outcomes including patient-reported outcomes.
c) The datasets identified as part of this review will in future be used, along with other sources, to create a fit-for-purpose assessment to determine if real-world databases in diabetes are suitable to answer specific research questions.
13. Searches

A systematic search strategy was employed, across multiple databases, to identify real-world databases in diabetes. Searches will be conducted within MEDLINE, Cumulative Index to Nursing and Allied Health Literature (CINAHL), and EMBASE.

Finally, a list of 50 eminent researchers in the field of diabetes and real-world evidence will be contacted via email and asked to contribute any databases which they are aware of that may have been missed by the systematic search.

14. Search strategy

The search strategy will be developed and piloted within PubMed before being adapted for other databases. The PubMed search strategy is included below.

Ovid MEDLINE(R) ALL <1946 to September 29, 2023>

1	diabetes mellitus.mp.	536193

2	diabet*.ti,ab.	777104

3	drug prescription.mp.	2269

4	drug prescri*.ti,ab.	5424

5	drug therapy.mp.	2711198

6	drug therap*.ti,ab.	44907

7	medication.ti,ab.	270923

8	continuous glucose monitor*.ti,ab.	6713

9	insulin pump.ti,ab.	2726

10	mortaliy.mp.	17

11	mortality.ti,ab.	986007

12	cardiovascular.ti,ab.	548241

13	stroke.mp.	376763

14	stroke.ti,ab.	305676

15	diabetic nephropathies.mp.	29846

16	diabetic nephropath*.ti,ab.	21943

17	diabetic eye.ti,ab.	942

18	glycated hemoglobins.mp.	39

19	glycated hemoglobins.ti,ab.	35

20	glycated haemoglobins.ti,ab.	12

21	HbA1c.ti,ab.	46455

22	blood glucose.mp.	230443

23	blood glucose.ti,ab.	87069

24	outcomes.ti,ab.	1439864

25	quality of life.ti,ab.	377611

26	depression.ti,ab.	419237

27	patient reported.ti,ab.	54794

28	diabetes distress.ti,ab.	1014

29	fear of hypoglycaemia.ti,ab.	195

30	fear of hypoglycemia.ti,ab.	387

31	real-world data*.ti,ab.	11318

32	database.ti,ab.	436312

33	electronic health record*.ti,ab.	27610

34	registr*.ti,ab.	361129

35	longitudinal.ti,ab.	328484

36	retrospective.ti,ab.	762205

37	prospective.ti,ab.	725191

38	exp animals/ not humans.sh.	5159300

39	infant, newborn.mp.	672982

40	infant.mp.	1321350

41	child, preschool.mp.	992123

42	Adolescent.mp.	2264528

43	child.mp.	2385351

44	1 or 2	826105

45	3 or 4 or 5 or 6 or 7 or 8 or 9	2893620

46	10 or 11 or 12 or 13 or 14 or 15 or 16 or 17 or 18 or 19 or 20 or 21 or 22 or 23 or 24 or 25 or 26 or 27 or 28 or 29 or 30	3689627

47	31 or 32 or 33 or 34 or 35 or 36 or 37	2359890

48	39 or 40 or 41 or 42 or 43	4192950

49	44 and 45 and 46 and 47	17975

50	49 not 38 not 48	15394

15. Condition or domain being studied

RWD databases which have collected diabetes related data including demographics, biochemistry, multi-morbidity, therapies, lifestyle data including continuous glucose monitoring and outcomes such as patient-reported outcomes.

16. Participants/population

Adults (aged over 18 years) who have been diagnosed with either Type 1 or Type 2 diabetes mellitus.

17. Exposure(s)

This systematic review will not assess an exposure.

18. Comparators/controls

No comparator or control are required for inclusion.

19. Type of study to be included

Any RWD database will be included within the review including longitudinal datasets. Trials based studies, protocols, and reviews will not be included in the final review.

20. Main outcomes

The main outcome of the review is to identify RWD databases which have collected data regarding diabetes. Meta-data regarding each included database will be extracted. From the information collected, a series of fit-for-purpose considerations will be derived to allow future researchers to identify if a RWD database is suitable to answer their specific research question.

21. Additional outcomes

All meta-data regarding each database will be made publicly available upon publication of the review. A website will be produced to house the datasets and related meta-data.

22. Data extraction

Once searches have been completed, all retrieved references will be imported into EndNote and duplicates will be removed. Then, two researchers will perform initial title and abstract screening. Any disagreements regarding inclusion of a study will be resolved via discussion between the two researchers. Following this, included articles will be full text screened by a single researcher.

All studies will be screened based on the inclusion criteria outlined in Table 1 below.

Table S1. Inclusion and exclusion criteria used for screening.

| **Inclusion** | **Exclusion** |
| --- | --- |
| Describe or analyse a RWD database which focuses on patients with diabetes * | Focuses on a paediatric or gestational population |
| Observational, longitudinal study design; or include routinely collected health data | Does not focus on a population living with diabetes (either type 1 or type 2) |
| Contain data on at least 1000 participants | Cross-sectional study design (data only available at one time point) |
| Include a follow-up duration of at least 12 months | Is a systematic review or protocol paper* |
| Published in last 10 years* |  |

* Criteria applied to the publication, not the dataset

Data extraction will then be completed in two stages by a single researcher on included studies. Firstly, data regarding the papers identified as part of the review will be extracted including date of publication, dataset name. Secondly, data regarding the datasets will be extracted including the number of participants included country/countries of data collection, average age of individuals in the dataset, and variables held within the dataset such as mortality outcomes or biochemical variables. A standardised data extraction form will be used at both stages of this process and will be uploaded once finalised.

23. Risk of bias (quality) assessment

As the review aims to identify RWD datasets, rather than evaluating the results of specific studies, no risk of bias or quality assessment will be conducted.

24. Data synthesis

Once data has been extracted from each dataset it will be formatted into a table to highlight the key characteristics of each dataset. Key patterns and trends in datasets will then be obtained. Basic summary statistics will be produced to describe these relationships such as the proportion of datasets collected in given geographic areas, number of participants per dataset, and age range across datasets. Visualisations will also be produced to further synthesis this information and improve its understandability.

25. Analysis of sub-groups and sub-sets

Datasets may be sub-divided for some descriptives. For example, by country of data collection, or by type of exposure (GLP-1, SGLT2, insulin, etc).

26. Type of method and review

Systematic review

27. Language

English

28. Country

England

29. Other registration details

This review is not registered elsewhere.

30. Reference and/or URL for published protocol

None

31. Dissemination plan

The outcomes of this review will be published in a peer-reviewed journal. Results will also be presented to collaborators on the REDDIE project as well as presented at conferences. A website will also be created to house the datasets and relevant meta-data related to each dataset.

32. Keywords

Diabetes,

33. Details of any existing review of the same topic by the same author

Not applicable

34. Current review status

In-progress

35. Any additional information

None

36. Details of final report/publication(s) or preprints if available

Currently not applicable. Will be added once available.

**References**

1.	Saeedi P, Petersohn I, Salpea P, Malanda B, Karuranga S, Unwin N, et al. Global and regional diabetes prevalence estimates for 2019 and projections for 2030 and 2045: Results from the International Diabetes Federation Diabetes Atlas, 9<sup>th</sup> edition. Diabetes Research and Clinical Practice. 2019;157.

2.	Amr Makady and Anthonius and Hans Hillege and Olaf Klungel and Wim G. What Is Real-World Data? A Review of Definitions Based on Literature and Stakeholder Interviews. Value in Health. 2017;20(7):858-65.

3.	Administration USFaD. Real world evidence 2018 [Available from: <https://www.fda.gov/science-research/science-and-research-special-topics/real-world-evidence>.

4.	Briana Coles and Freya Tyrer and Humaira Hussein and Nafeesa Dhalwani and Kamlesh K. Development, content validation, and reliability of the Assessment of Real-World Observational Studies (ArRoWS) critical appraisal tool. Annals of Epidemiology. 2021;55:57-63.e15.

Electronic Supplementary Material 2: Preferred reporting items in systematic reviews and meta-analysis (PRISMA) statement:

| **Section and Topic** | **Item #** | **Checklist item** | **Location where item is reported** |
| --- | --- | --- | --- |
| **TITLE** | | |  |
| Title | 1 | Identify the report as a systematic review. | 1 |
| **ABSTRACT** | | |  |
| Abstract | 2 | See the PRISMA 2020 for Abstracts checklist. |  |
| **INTRODUCTION** | | |  |
| Rationale | 3 | Describe the rationale for the review in the context of existing knowledge. | 2-4 |
| Objectives | 4 | Provide an explicit statement of the objective(s) or question(s) the review addresses. | 4 |
| **METHODS** | | |  |
| Eligibility criteria | 5 | Specify the inclusion and exclusion criteria for the review and how studies were grouped for the syntheses. | 5 |
| Information sources | 6 | Specify all databases, registers, websites, organisations, reference lists and other sources searched or consulted to identify studies. Specify the date when each source was last searched or consulted. | 5 |
| Search strategy | 7 | Present the full search strategies for all databases, registers and websites, including any filters and limits used. | Supplementary material 1 |
| Selection process | 8 | Specify the methods used to decide whether a study met the inclusion criteria of the review, including how many reviewers screened each record and each report retrieved, whether they worked independently, and if applicable, details of automation tools used in the process. | 6 |
| Data collection process | 9 | Specify the methods used to collect data from reports, including how many reviewers collected data from each report, whether they worked independently, any processes for obtaining or confirming data from study investigators, and if applicable, details of automation tools used in the process. | 6-7 |
| Data items | 10a | List and define all outcomes for which data were sought. Specify whether all results that were compatible with each outcome domain in each study were sought (e.g. for all measures, time points, analyses), and if not, the methods used to decide which results to collect. | 6-7 |
|  | 10b | List and define all other variables for which data were sought (e.g. participant and intervention characteristics, funding sources). Describe any assumptions made about any missing or unclear information. | 6-7 |
| Study risk of bias assessment | 11 | Specify the methods used to assess risk of bias in the included studies, including details of the tool(s) used, how many reviewers assessed each study and whether they worked independently, and if applicable, details of automation tools used in the process. | NA |
| Effect measures | 12 | Specify for each outcome the effect measure(s) (e.g. risk ratio, mean difference) used in the synthesis or presentation of results. | NA |
| Synthesis methods | 13a | Describe the processes used to decide which studies were eligible for each synthesis (e.g. tabulating the study intervention characteristics and comparing against the planned groups for each synthesis (item #5)). | 6-7 |
|  | 13b | Describe any methods required to prepare the data for presentation or synthesis, such as handling of missing summary statistics, or data conversions. | 6-7 |
|  | 13c | Describe any methods used to tabulate or visually display results of individual studies and syntheses. | 6-7 |
|  | 13d | Describe any methods used to synthesize results and provide a rationale for the choice(s). If meta-analysis was performed, describe the model(s), method(s) to identify the presence and extent of statistical heterogeneity, and software package(s) used. | NA |
|  | 13e | Describe any methods used to explore possible causes of heterogeneity among study results (e.g. subgroup analysis, meta-regression). | NA |
|  | 13f | Describe any sensitivity analyses conducted to assess robustness of the synthesized results. | NA |
| Reporting bias assessment | 14 | Describe any methods used to assess risk of bias due to missing results in a synthesis (arising from reporting biases). | NA |
| Certainty assessment | 15 | Describe any methods used to assess certainty (or confidence) in the body of evidence for an outcome. | NA |
| **RESULTS** | | |  |
| Study selection | 16a | Describe the results of the search and selection process, from the number of records identified in the search to the number of studies included in the review, ideally using a flow diagram. | NA |
|  | 16b | Cite studies that might appear to meet the inclusion criteria, but which were excluded, and explain why they were excluded. | NA |
| Study characteristics | 17 | Cite each included study and present its characteristics. | Online dashboard |
| Risk of bias in studies | 18 | Present assessments of risk of bias for each included study. | NA |
| Results of individual studies | 19 | For all outcomes, present, for each study: (a) summary statistics for each group (where appropriate) and (b) an effect estimate and its precision (e.g. confidence/credible interval), ideally using structured tables or plots. | 8-12 |
| Results of syntheses | 20a | For each synthesis, briefly summarise the characteristics and risk of bias among contributing studies. | NA |
|  | 20b | Present results of all statistical syntheses conducted. If meta-analysis was done, present for each the summary estimate and its precision (e.g. confidence/credible interval) and measures of statistical heterogeneity. If comparing groups, describe the direction of the effect. | NA |
|  | 20c | Present results of all investigations of possible causes of heterogeneity among study results. | NA |
|  | 20d | Present results of all sensitivity analyses conducted to assess the robustness of the synthesized results. | NA |
| Reporting biases | 21 | Present assessments of risk of bias due to missing results (arising from reporting biases) for each synthesis assessed. | NA |
| Certainty of evidence | 22 | Present assessments of certainty (or confidence) in the body of evidence for each outcome assessed. | NA |
| **DISCUSSION** | | |  |
| Discussion | 23a | Provide a general interpretation of the results in the context of other evidence. | 12-15 |
|  | 23b | Discuss any limitations of the evidence included in the review. | 15-16 |
|  | 23c | Discuss any limitations of the review processes used. | 15-16 |
|  | 23d | Discuss implications of the results for practice, policy, and future research. | 12-16 |
| **OTHER INFORMATION** | | |  |
| Registration and protocol | 24a | Provide registration information for the review, including register name and registration number, or state that the review was not registered. | 4 |
|  | 24b | Indicate where the review protocol can be accessed, or state that a protocol was not prepared. | 4 |
|  | 24c | Describe and explain any amendments to information provided at registration or in the protocol. | 4 |
| Support | 25 | Describe sources of financial or non-financial support for the review, and the role of the funders or sponsors in the review. | 16-17 |
| Competing interests | 26 | Declare any competing interests of review authors. | 17-18 |
| Availability of data, code and other materials | 27 | Report which of the following are publicly available and where they can be found: template data collection forms; data extracted from included studies; data used for all analyses; analytic code; any other materials used in the review. | 17-18 |

*From:* Page MJ, McKenzie JE, Bossuyt PM, Boutron I, Hoffmann TC, Mulrow CD, et al. The PRISMA 2020 statement: an updated guideline for reporting systematic reviews. BMJ 2021;372:n71. doi: 10.1136/bmj.n71

Electronic Supplementary Material 3: Search Strategy

OVID MEDLINE

Patient

1. diabet*.ti,ab.
2. diabetes mellitus.mp.

Exposures

3. drug prescriptions.mp.
4. drug prescri*.ti,ab.
5. drug therapy.mp.
6. drug therap*.ti,ab.
7. medication.ti,ab.

Outcomes

8. mortality.mp.
9. cardiovascular.ti,ab.
10. stroke.mp.
11. stroke.ti,ab.
12. diabetic nephropathies.mp.
13. diabetic nephropath*.ti,ab.
14. diabetic eye.ti,ab.
15. glycated hemoglobins.mp.
16. glycated hemoglobins.ti,ab.
17. glycated haemoglobins.ti,ab.
18. HbA1c.ti,ab.
19. blood glucose.mp
20. outcomes.ti,ab.
21. quality of life.ti,ab.
22. depression.ti,ab.
23. patient reported.ti,ab.
24. diabetes distress.ti,ab.
25. fear of hypoglycaemia.ti,ab.

Study type

26. real world.ti,ab.
27. real-world.ti,ab.
28. real world data*.ti,ab.
29. real-world data*.ti,ab.
30. epidemiologic stud*.ti,ab.
31. case control stud*.ti,ab.
32. cohort stud*.ti,ab.
33. cohort studies.mp.
34. cohort analy*.ti,ab.
35. follow up stud*.ti,ab.
36. follow-up stud*.ti,ab.
37. observational stud*.ti,ab.
38. longitudinal.ti,ab.
39. retrospective.ti,ab.
40. cross-sectional.ti,ab.
41. cross-sectional.ti,ab.
42. 1 or 2
43. 3 or 4 or 5 or 6 or 7
44. 8 or 9 or 10 or 11 or 12 or 13 or 14 or 15 or 16 or 17 or 18 or 19 or 20 or 21 or 22 or 23 or 24 or 25
45. 26 or 27 or 28 or 29 or 30 or 31 or 32 or 33 or 34 or 35 or 36 or 37 or 38 or 39 or 40 or 41
46. 42 and 43 and 44 and 45
47. exp animals/ not humans.sh.
48. infant, newborn.mp.
49. infant.mp.
50. child, preschool.mp.
51. Adolescent.mp.
52. child.mp.
53. 48 or 49 or 50 or 51 or 52
54. 46 not 47 not 53

**SCOPUS**

( KEY ( diabetes AND mellitus ) OR TITLE ( diabet* ) OR ABS ( diabet* ) )

( KEY ( drug AND prescription ) OR TITLE ( drug AND prescri* ) OR ABS ( drug AND prescri* ) OR KEY ( drug AND therapy ) OR TITLE ( drug AND therap* ) OR ABS ( drug AND therap* ) OR TITLE ( medication ) OR ABS ( medication ) )

( KEY ( mortality ) OR TITLE ( cardiovascular ) OR ABS ( cardiovascular ) OR KEY ( stroke ) OR TITLE ( stroke ) OR ABS ( stroke ) OR KEY ( diabetic AND nephropathies ) OR TITLE ( diabetic AND nephropath* ) OR ABS ( diabetic AND nephropath* ) OR TITLE ( diabetic AND eye ) OR ABS ( diabetic AND eye ) OR KEY ( glycated AND hemoglobins ) OR TITLE ( glycated AND hemoglobins ) OR ABS ( glycated AND hemoglobins ) OR TITLE ( glycated AND haemoglobins ) OR ABS ( glycated AND haemoglobins ) OR TITLE ( hba1c ) OR ABS ( hba1c ) OR KEY ( blood AND glucose ) OR TITLE ( outcomes ) OR ABS ( outcomes ) OR TITLE ( quality AND of AND life ) OR ABS ( quality AND of AND life ) OR TITLE ( depression ) OR ABS ( depression ) OR TITLE ( patient AND reported ) OR ABS ( patient AND reported ) OR TITLE ( diabetes AND distress ) OR ABS ( diabetes AND distress ) OR TITLE ( fear AND of AND hypoglycaemia ) OR ABS ( fear AND of AND hypoglycaemia ) )

( TITLE ( real AND world ) OR ABS ( real AND world ) OR TITLE ( real-world ) OR ABS ( real-world ) OR TITLE ( real AND world AND data ) OR ABS ( real AND world AND data ) OR TITLE ( real-world AND data ) OR ABS ( real-world AND data ) OR TITLE ( epidemiologic AND stud* ) OR ABS ( epidemiologic AND stud* ) OR TITLE ( case AND control AND stud* ) OR ABS ( case AND control AND stud* ) OR TITLE ( cohort AND stud* ) OR ABS ( cohort AND stud* ) OR KEY ( cohort AND studies ) OR TITLE ( cohort AND analysis ) OR ABS ( cohort AND analysis ) OR TITLE ( follow AND up AND stud* ) OR ABS ( follow AND up AND stud* ) OR TITLE ( follow-up AND stud* ) OR ABS ( follow-up AND stud* ) OR TITLE ( observational AND stud* ) OR ABS ( observational AND stud* ) OR TITLE ( longitudinal ) OR ABS ( longitudinal ) OR TITLE ( retrosepctive ) OR ABS ( retrospective ) OR TITLE ( cross AND sectional ) OR ABS ( cross AND sectional ) OR TITLE ( cross-sectional ) OR ABS ( cross-sectional ) )

( KEY ( diabetes AND mellitus ) OR TITLE ( diabet* ) OR ABS ( diabet* ) ) AND ( KEY ( drug AND prescription ) OR TITLE ( drug AND prescri* ) OR ABS ( drug AND prescri* ) OR KEY ( drug AND therapy ) OR TITLE ( drug AND therap* ) OR ABS ( drug AND therap* ) OR TITLE ( medication ) OR ABS ( medication ) ) AND ( KEY ( mortality ) OR TITLE ( cardiovascular ) OR ABS ( cardiovascular ) OR KEY ( stroke ) OR TITLE ( stroke ) OR ABS ( stroke ) OR KEY ( diabetic AND nephropathies ) OR TITLE ( diabetic AND nephropath* ) OR ABS ( diabetic AND nephropath* ) OR TITLE ( diabetic AND eye ) OR ABS ( diabetic AND eye ) OR KEY ( glycated AND hemoglobins ) OR TITLE ( glycated AND hemoglobins ) OR ABS ( glycated AND hemoglobins ) OR TITLE ( glycated AND haemoglobins ) OR ABS ( glycated AND haemoglobins ) OR TITLE ( hba1c ) OR ABS ( hba1c ) OR KEY ( blood AND glucose ) OR TITLE ( outcomes ) OR ABS ( outcomes ) OR TITLE ( quality AND of AND life ) OR ABS ( quality AND of AND life ) OR TITLE ( depression ) OR ABS ( depression ) OR TITLE ( patient AND reported ) OR ABS ( patient AND reported ) OR TITLE ( diabetes AND distress ) OR ABS ( diabetes AND distress ) OR TITLE ( fear AND of AND hypoglycaemia ) OR ABS ( fear AND of AND hypoglycaemia ) ) AND ( TITLE ( real AND world ) OR ABS ( real AND world ) OR TITLE ( real-world ) OR ABS ( real-world ) OR TITLE ( real AND world AND data ) OR ABS ( real AND world AND data ) OR TITLE ( real-world AND data ) OR ABS ( real-world AND data ) OR TITLE ( database* ) OR ABS ( database* ) OR TITLE ( registr* ) OR ABS ( registr* ) OR TITLE ( longitudinal ) OR ABS ( longitudinal ) )

CINAHL

Patient/population

1. Diabetes Mellitus [MH]
2. Diabet* [ti]
3. Diabet* [ab]
4. 1 OR 2 OR 3

Exposure

5. Drug prescription [MH]
6. Drug prescri* [ti]
7. Drug prescri* [ab]
8. Drug therapy [MH]
9. Drug therap* [ti]
10. Drug therap* [ab]
11. Glycated hemoglobins [MH]
12. Glycated hemoglobins [ti]
13. Glycated hemoglobins [ab]
14. Glycated haemoglobins [ti]
15. Glycated haemoglobins [ab]
16. HbA1c [ti]
17. HbA1c [ab]
18. Blood glucose [MH]
19. 4 through 30 with OR

Outcome

20. Mortality [MH]
21. Cardiovascular mortality [ti]
22. Cardiovascular mortality [ab]
23. Stroke [MH]
24. Stroke [ti]
25. Stroke [ab]
26. Diabetic Nephropathies [MH]
27. Diabetic nephropath* [ti]
28. Diabetic nephropath* [ab]
29. Diabetic eye [ti]
30. Diabetic eye [ab]
31. 32 through 42 with OR

Study type

32. Real world evidence [ti]
33. Real world evidence [ab]
34. Real-world evidence [ti]
35. Real-world evidence [ab]
36. Real world data* [ti]
37. Real world data* [ab]
38. Real-world data* [ti]
39. Real-world data* [ab]
40. Epidemiologic stud* [ti]
41. Epidemiologic stud* [ab]
42. case control stud* [ti]
43. case control stud* [ab]
44. cohort stud* [ti]
45. cohort stud* [ab]
46. Cohort studies [MH]
47. Cohort analy* [ti]
48. Cohort analy* [ab]
49. Follow up stud* [ti]
50. Follow up stud* [ab]
51. Follow-up stud* [ti]
52. Follow-up stud* [ab]
53. Observational stud* [ti]
54. Observational stud* [ab]
55. Longitudinal [ti]
56. Longitudinal [ab]
57. Retrospective [ti]
58. Retrospective [ab]
59. Cross sectional [ti]
60. Cross sectional [ab]
61. Cross-sectional [ti]
62. Cross-sectional [ab]
63. 44 through 74 with OR

Filters

Animals

((MH "Vertebrates+") NOT MH Human)

Adult

No adult filter applied

Electronic Supplementary Material 4: Description of extracted variables:

| **Variable** | **Description** |
| --- | --- |
| Demographics | Reported any one of sex, ethnicity, age. |
| Prescription | Reported the medications prescribed or currently being taken. |
| Diagnosis | Reporting of ICD codes or reporting of previously diagnosed conditions. |
| Mortality | Reporting of any form of mortality. |
| Biochemistry | Reported any one of HbA1c, fasting glucose, blood lipids, |
| Technology | Reporting of use of diabetes technology including continuous glucose monitors or automated/semi-automated insulin delivery system. |
